# Elevated Phospholipid Transfer Protein in Subjects with Multiple Sclerosis

**DOI:** 10.1155/2015/518654

**Published:** 2015-08-12

**Authors:** Roy A. Garvin

**Affiliations:** BVBiomed Ltd., Oregon Bioscience Incubator, 4640 SW Macadam Avenue No. 200, Portland, OR 97239, USA

## Abstract

An anomaly in the plasma proteins of patients with multiple sclerosis detectable on SDS-PAGE has been reported. The molecular weight of the anomaly was the same as the phospholipid transfer protein. A metabolic protein was involved in lipid homeostasis and remodeling of the high density lipoproteins. We have identified the anomaly as the phospholipid transfer protein by western blot using antiphospholipid transfer antibodies. Activity assays showed that the phospholipid transfer activity was elevated in fasted plasma samples from subjects with MS compared to controls. Sequence analysis of the gene encoding the phospholipid transfer protein did not identify any mutations in the genetic structure, suggesting that the increase in activity was not due to structural changes in the protein, but may be due to one of the other proteins with which it forms active complexes. Altered phospholipid transfer activity is important because it could be implicated in the decreased lipid uptake and abnormal myelin lipids observed in multiple sclerosis. It has been shown that alteration in myelin lipid content is an epitope for autoimmunity. Therefore, lipid changes due to a defect in phospholipid transfer and/or uptake could potentially influence the course of the disease. Further research is needed to elucidate the role of the phospholipid transfer protein in subjects with multiple sclerosis.

## 1. Introduction

We have previously reported a protein anomaly in the plasma of fasted multiple sclerosis (MS) patients [1]. This anomaly has a molecular weight by SDS-PAGE of 70 kDa and is elevated in the plasma of MS patients whether fasted or fed but not in subjects without the disease. This responsiveness to food intake, in individuals with MS, suggests a metabolic role for the protein.

Alterations in metabolic pathways in subjects with MS have been suggested before [2]. These include abnormalities in the plasma proteins [3, 4], reduced levels of lecithin cholesterol acyltransferase in the brain [5], alterations in plasma [6, 7] and cellular lipid profiles [8, 9], and differences in the phospholipid content of normal myelin compared with myelin from subjects with MS [10].

One group of metabolic proteins of particular interest is the plasma lipid transfer proteins which include the cholesterol ester transfer protein (CETP) and phospholipid transfer protein (PLTP) [11]. CETP transfers cholesteryl esters (CE) and triglycerides (TG) between lipoproteins. PLTP transfers phospholipids but lacks the ability to transfer either CE or TG. Both CETP and PLTP have been detected in plasma and cerebrospinal fluid (CSF) [12, 13] where they play important roles in maintaining lipid homeostasis. In this study we identified the plasma abnormality as PLTP and characterized it in plasma from subjects with MS.

## 2. Methods

### 2.1. Patients

Blood samples were taken from individuals who had been fasted for more than eight hours. There were 111 MS subjects: 38 male and 73 female, with a mean age of 38, a mean Kurtzke [14] score of 5.2, and a confirmed diagnosis of MS. Sixty of the MS patients were on a low fat diet alone, 3 were on a low fat diet and steroids, 5 were on steroids alone, and 47 of those on dietary treatment received supplements of polyunsaturated fatty acids. In addition there were 45 controls, comprising 30 individuals with no disease and 15 subjects with diseases other than MS (12 with lipid disorders, 5 with neurological disease, and 2 with other diseases). Samples from patients with demyelinating diseases were included to eliminate the possibility that the plasma protein or its activity was related to the process of demyelination.

### 2.2. Sample Collection

Whole blood was obtained by venepuncture in 5 mL vacutainers containing 0.05% EDTA as anticoagulant (Beckton-Dickinson). Whole blood samples were frozen at −80°C prior to DNA isolation. Plasma was obtained by centrifugation (5000 rpm for 15 minutes) and held at −80°C until analyzed.

### 2.3. Lipid Transfer Assays

The ability of plasma from MS patients and controls to transfer labeled lipid substrate from VLDL to HDL was compared. The assays were carried out as described by Tollefson and Albers [15] except that the lipoproteins were separated in the last step by phosphotungstic acid/magnesium chloride precipitation of the HDL [16]. Briefly, lipoprotein donors (very low density lipoproteins (VLDL)) and acceptors (high density lipoproteins (HDL)) were prepared by ultracentrifugation. The substrate ^3^H-phosphatidyl choline (PC) was obtained from New England Nuclear, Boston, Mass. It was incorporated by exchange into the donor lipoproteins; that is, radiolabelled lipid was added to purified VLDL and allowed to equilibrate with the lipids present during a 3 hr incubation at 37°C.

For each batch of acceptor and donor lipoproteins the conditions of the assay were optimized with respect to the donor/acceptor ratio, the amount of transfer factor added, and the time of incubation. This was done by holding the amount of added acceptor constant and varying either the amount of donor or transfer activity added, or the time of incubation. At the optimum acceptor to donor ratio (1 : 5 VLDL/PC-HDL, 1 : 20 VLDL/CE-HDL, and 1 : 50 VLDL/TG-HDL) with an incubation time of 3 hr at 37°C and 10 *μ*L of added transfer activity (plasma sample) the assay was linear. The final steps were to precipitate the VLDL and then transfer 500 *μ*L of supernatant (HDL) to 5 mL of scintillant. This mixture was counted for 10 minutes, or to <2% SD. Concurrent assays done as above but incubated at 0°C showed no appreciable transfer of PC.

Since PLTP is heat labile and will lose all of its transfer activity if held at 65°C for 60 minutes, the transfer assay was carried out on untreated plasma samples and heat treated aliquots (no PLTP activity). In this way we were able to determine the amount of active transport due solely to PLTP. The transfer of PC due solely to PLTP could then be calculated as (CPM^HDL  untreated^ − CPM  HDL^heat  treated^/total  CPM/assay) × 100.

### 2.4. Western Blot Analysis

Gel electrophoresis and western blot analysis were carried out as described by Mahmood and Yang [17]. Primary anti-PLTP antibodies were kindly provided by Dr. John Albers, Department of Medicine, Northwest Lipid Metabolism and Diabetes Research Laboratories, University of Washington, Seattle, Washington 98109-4517, USA.

### 2.5. Genomic DNA Isolation

Genomic DNA isolation was done using the QIAamp DNA Blood Mini Kit (QIAGEN GmbH Hilden, Germany) according to the manufacturer's directions.

### 2.6. PCR Amplification of PLTP from Genomic DNA

PCR amplification of PLTP was performed as described by primers pairs selected from the published genomic DNA sequence (see Table 1). Genomic amplification for all sets of primers was optimised for MgCl_2_ and annealing temperature, and addition of DMSO was used to stop nonspecific binding. The amplification mixture used for all of the primers was 10 *μ*L of 10x reaction buffer, 5 *μ*L DMSO, 1 *μ*L of dNTPs, 1 *μ*M of each primer, 6 *μ*L of MgCl_2_ stock solution, 10 *μ*L of genomic DNA (50–100 ng), and 0.5 *μ*L of Taq polymerase. Amplification was done using the following conditions: 95°C for 2 minutes and then 72°C for 2 minutes. This was followed by 35 cycles of 95°C for 1 minute, 56°C or 60°C for 1 minute, and 72°C for 1 minute, with a final extension at 72°C for 5 minutes.

### 2.7. Sequencing of PLTP

Sequencing of PLTP from genomic DNA was done using the Sanger method [18, 19] and ABI 310 DNA sequencer (Thermo Fisher Scientific, Grand Island, NY, USA).

## 3. Results

**Figure 1:**
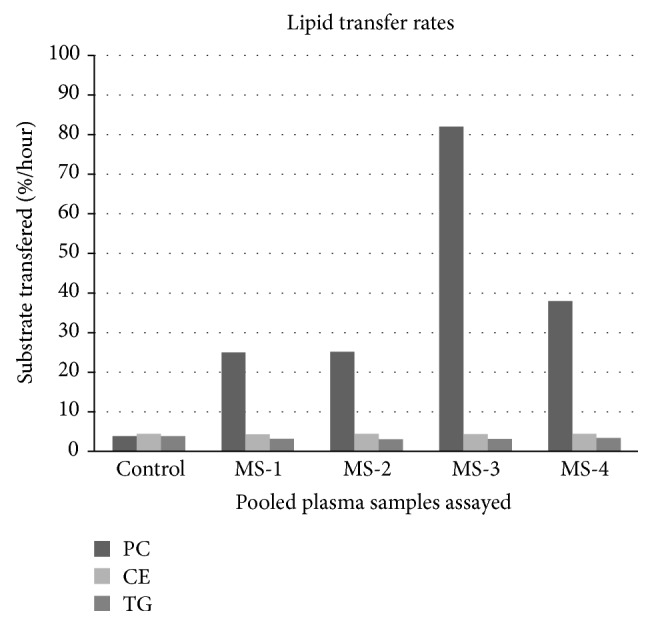
Lipid transfer rates for pooled plasma samples represented as percent substrate transferred per hour. PLTP activity showed an 8-fold increase in average transfer rates. Triglycerides showed a 19% decrease in transfer by MS samples. There was no diference in cholesterol transfer between the groups.

Measurements of CETP activity in 4 batches of pooled plasma from MS patients (*n* = 6 per pool) and a pool of healthy controls (*n* = 6) showed an increase in the ability of plasma from MS patients to transfer phosphatidyl choline from VLDL to HDL (i.e., PLTP activity, see Figure 1 and supplemental data in the Supplementary Material available online at http://dx.doi.org/10.1155/2015/518654). There were no significant differences in the transfer of cholesterol esters, although the MS samples showed a decrease in triglycerides transfer (−19%).

**Figure 2:**
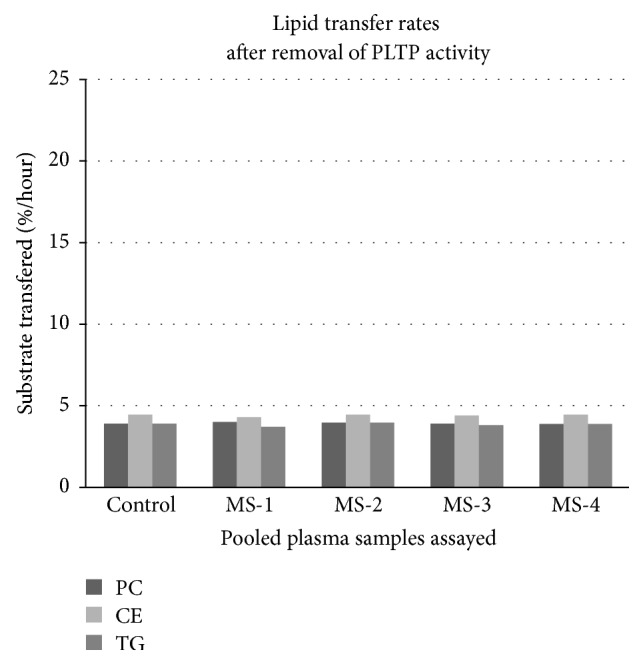
Lipid transfer rates for pooled plasma samples following heat treatment to remove PLTP activity. Results are expressed as percent substrate transferred per hour.

When these assays were repeated using aliquots of plasma which had been heat treated, almost all of the PLTP activity had been removed (see Figure 2) suggesting that the identity of the protein anomaly in MS plasma could be PLTP. This was confirmed by western blot analysis using anti-PLTP antibodies (data not shown).

**Figure 3:**
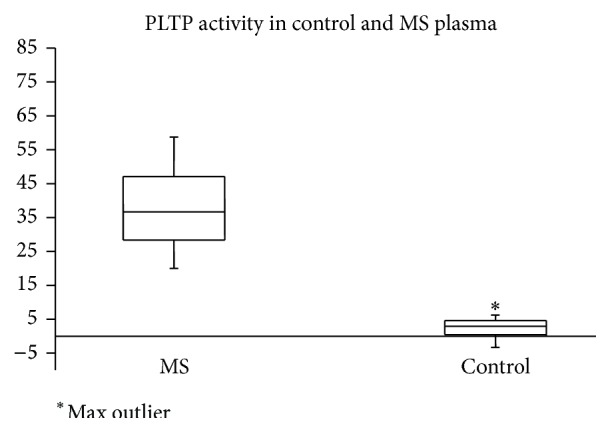
Plasma samples analyzed as “blind” for PLTP activity. Results are expressed as percent substrate transferred per minute. The subjects with MS all had elevated PLTP activity (average = 36.5, range = 20.2–56.7%). One control had an elevated PLTP activity similar to that seen in MS.

A range for PLTP activity in subjects with MS and controls was established in plasma samples from 29 controls and 33 subjects with MS. The PLTP activity of MS patients was >20% PC/hr while controls transferred <4% PC/hr (supplemental data). PLTP activity was then measured blind in MS and control samples using the HDL/VLDL assay (38 MS and 15 control, Figure 3 and supplemental data). The MS subjects all had elevated plasma PLTP activity (average = 36.5, range = 20.2–56.7%). One control sample also had elevated PLTP activity similar to that seen in MS.

The exons encoding PLTP were then sequenced from subjects with MS and controls. When compared to the published sequence, no mutations or alterations were identified in the DNA of subjects with MS.

## 4. Discussion

This study identified a plasma abnormality in subjects with MS as PLTP. It was demonstrated that plasma samples from fasted subjects with MS showed a marked increase in ability to transfer phospholipids between the lipoproteins, HDL and VLDL, when compared to matched controls. Interestingly, samples from the MS subjects did not show any change in their ability to transfer either cholesterol esters or triacylglycerides mediated by CETP nor was any change in PLTP activity seen in plasma samples from patients with Guillain Barré Syndrome with active demyelination, suggesting that the increased lipid transfer activity seen in the plasma of MS subjects was not linked to myelin debris accumulation.

In contrast, PLTP activity has been reported to be decreased in the CSF of subjects with MS [20]. And the decrease in CSF PLTP activity was shown to be dependent on the level of lathosterol, a precursor of HDL. If PLTP activity in the plasma is also dependent on the level of lathosterol level, then this may explain the elevation of PLTP activity observed in the plasma of subjects with MS. Since lathosterol levels are known to be increased in the CSF of MS patients [21] but decreased in the plasma [22], elevated PLTP activity in the plasma could also explain the decreased levels of HDL-C reported in MS [23] since plasma HDL-C levels are inversely associated with PLTP activity [24].

Genetic analysis of the gene encoding PLTP did not detect any changes in the DNA which might alter the structure and affect the activity of PLTP, suggesting that the increase in PLTP activity in the plasma could be due to other forces. This could be due to the decreased lathosterol levels in the plasma. Alternatively, it could be due to alterations in one of the cofactors with which PLTP forms complexes. These include Apo E and Apo-A-1 [25] which can affect PLTP specific activity. Other complexing proteins are linked to immunity, inflammation, and the complement and coagulation cascades [26] which may also affect PLTP activity.

## 5. Conclusions

Although the cause remains unclear, altered PLTP activity in the CSF and plasma is an important area of research in MS. As it could contribute either directly or indirectly to changes in the phospholipid content which have been described in the plasma, cellular membranes, and myelin of subjects with MS, these changes in lipid homeostasis are potentially significant because alterations in myelin lipids promote epitope binding and antibody formation [27]. In addition, the demyelination which is characteristic of the disease is preceded by inflammation and an autoimmune response, which can be regulated by changes in the lipid subfractions. And lastly, the subclasses of HDL which are regulated by PLTP [28] play a role in maintaining the blood brain barrier (BBB) [29].

Further work is needed to understand the underlying mechanisms responsible for the alterations in PLTP activity observed in the CSF and plasma of subjects with MS and the cause of the different levels of PLTP activity observed between the CSF and plasma. Since PLTP activity is linked to inflammation, autoimmunity, and the stability of the BBB, mechanisms affecting PLTP activity may provide new targets of research in MS.

## Supplementary Material

Tables show the ability of pooled plasma samples to transfer PC, CE and TG between HDL and VLDL. And the range of PC transfer activity measured in subjects with MS and controls. The controls included healthy subjects, neurological diseases other than MS, lipid disorders and other diseases. These results showed a marked increase in the PC transfer activity in the plasma of subjects with MS. Results of blind assay are presented in table format which shows the accuracy of identifying MS subjects based on plasma PC transfer activity.

## Figures and Tables

**Table 1:** Genomic primer pairs.

Primer	Direction	Sequence	Melting temp. °C	Annealing temp. °C	Exon size
Exon 5	Forward	GAG TGA ATA TTA ACC CCC CTG	62.0	60.0	107
Exon 5	Reverse	AGC TGG GGT TGG GGC TGG	62.0	60.0	
Exon 10	Forward	CCT CAC TCC TGA TTC CCC TG	64.0	62.0	63
Exon 10	Reverse	TAT CCC TGC CCC CGC CAG	64.0	62.0	
Exon 12	Forward	GAA GCT GGA GCT GCG GGT C	57.4	53.0	304
Exon 12	Reverse	CAG GTC CAG CTG CGT GCG CA	59.9	53.0	
