# Supplementary material for: Elevated Phospholipid Transfer Protein in Subjects with Multiple Sclerosis
Source: J Lipids. 2015 Aug 12;2015:518654. doi: 10.1155/2015/518654 (PMC4549613; doi:10.1155/2015/518654)
Supplement: Supplementary file 1 — Tables show the ability of pooled plasma samples to transfer PC, CE and TG between HDL and VLDL. And the range of PC transfer activity measured in subjects with MS and controls. The controls included healthy subjects, neurological diseases other than MS, lipid disorders and other diseases. These results showed a marked increase in the PC transfer activity in the plasma of subjects with MS. Results of blind assay are presented in table format which shows the accuracy of identifying MS subjects based on plasma PC transfer activity. [file 518654.f1.pdf]

Supplemental Data

Lipid Transfer Acitivity

Sample	PC	CE	TG
Control	0.78	0.89	0.78
MS-1	1.25	0.86	0.64
MS-2	1.26	0.89	0.61
MS-3	4.1	0.88	0.63
MS-4	1.9	0.89	0.68

Percent lipid substrate transferred per minute by pooled plasma samples from subjects with MS and controls. Phospholipid transfer activity was elevated in MS (mean=1.58%, Range=1.25-4.10%) over the control group (0.78%). Cholesteral transfer was similar in MS (mean=0.885%, range=0.86-0.89%) as controls (0.89%). Triglyceride transfer was decreased in MS (0.638%, range=0.61-0.68%) compared to

controls (0.78%).

Phospholipid Transfer Ranges

Diagnosis	n	% Transfer (Ave)	Range	SD

Healthy Controls	10	0.8	(-3) - 4	2.3
Lipid Disorders	12	0.6	(-1) - 3	1.3
Neurological Diseases	5	2.1	1 - 4	1.0
Other Diseases	2	3.0	(1) - (5)	na
**Total Controls**	**29**	**1.1**	**(-5) - 4**	**1.9**
**Multiple Sclerosis**	**33**	**33.4**	**22 - 46**	**6.6**

PC transfer acitivities for MS, normal controls and other diseases: Expressed as % PC labelled HDL transfered/1 hour assay at 37^0C by 10µl of plasma. Data used to calculate reference ranges for plasma phospholipid transfer in both MS patients and controls. Subjects with MS showed increased ability to transfer PC from HDL.

Determination of PLTP Activiy

‘Assayed Blind’

Sample#	% Transfer	Assay diagnosis	Clinical diagnosis	Correct(yes/no)
1	2.00	Control	Control	YES
2	34.50	MS	MS	YES
3	25.00	MS	MS	YES
4	46.90	MS	MS	YES
5	0.00	Control	Control	YES
6	1.50	Control	Control	YES
7	29.60	MS	MS	YES
8	34.30	MS	MS	YES
9	-2.40	Control	Control	YES
10	47.50	MS	MS	YES
11	3.10	Control	Control	YES
12	27.00	MS	MS	YES
13	5.70	Control	Control	YES
**14**	**22.30**	**MS**	**Control**	**NO**
15	34.50	MS	MS	YES
16	56.70	MS	MS	YES
17	28.90	MS	MS	YES
18	20.20	MS	MS	YES
19	34.70	MS	MS	YES
20	3.40	Control	Control	YES
21	31.40	MS	MS	YES
22	45.60	MS	MS	YES
23	51.30	MS	MS	YES
24	28.00	MS	MS	YES

25	0.50	Control	Control	YES
26	36.70	MS	MS	YES
27	41.30	MS	MS	YES
28	39.40	MS	MS	YES
29	0.00	Control	Control	YES
30	52.30	MS	MS	YES
31	27.60	MS	MS	YES
32	-4.00	Control	Control	YES
33	21.50	MS	MS	YES
34	26.90	MS	MS	YES
35	38.40	MS	MS	YES
36	24.70	MS	MS	YES
37	39.40	MS	MS	YES
37	-3.40	Control	Control	YES
38	46.50	MS	MS	YES
39	35.40	MS	MS	YES
40	46.90	MS	MS	YES
41	0.40	Control	Control	YES
42	0.00	Control	Control	YES
43	32.70	MS	MS	YES
44	33.80	MS	MS	YES
45	31.00	MS	MS	YES
46	27.50	MS	MS	YES
47	2.60	Control	Control	YES
48	1.40	Control	Control	YES
49	41.50	MS	MS	YES
50	28.90	MS	MS	YES
51	35.90	MS	MS	YES
52	41.70	MS	MS	YES
53	43.70	MS	MS	YES
